# 

**Published:** 1878-04-20

**Authors:** 


					﻿fV?
r
02
c
o
5
ss
o
r*
O
pH
*5
HR
2?
X
o
•“5
CD
pH
fcl
o
3D
■H
rH
<
5
E-i
Eh
z;
□D
*>»
2-i
rn
X
<•
rH
VOL. VIII.	APRIL 20, 1878.	-	No A.
r THE SOUTHERN
MEDICAL RECORD,
i	.	/
p. JAonthly jJoUBNAL. of J^actical ^Aedicine.
Terms; $2per annum in advance. Club tfates, 5 copies $8. Single copies 20c.
“ QUICQUID PB.ECIPIES ESTO BEE VIS.”
Q
W
S
a
i
a
H
•—i
O
ao
>*
I2J
U
u
>
Q
H
O
)—i
H
ft
QD
(X
o
i—i
L
0
H
Pl I
ESTABLISHED IN 1870.
ATLANTA, GA.:
Sunny South Steam Publishing Houbi:.
Address R. C. WORD, 51. D., Managing Editor, Atlanta, Ga.
				

